# Ceramidase is involved in metabolic reprogramming of fibroblasts under hypoxia conditions

**DOI:** 10.3389/fphys.2026.1914022

**Published:** 2026-09-30

**Authors:** Michela Terlizzi, Emmanuel D’Andria, Chiara Colarusso, Simone Di Caprio, Anna Falanga, Maria Rosaria Lauro, Rosario Salvi, Rosalinda Sorrentino

**Affiliations:** 1 Laboratory of Applied Physiology and Pharmacology, Department of Pharmacy, DIFARMA, University of Salerno, Fisciano, Italy; 2 PhD Program in Drug Discovery and Development, Department of Pharmacy, DIFARMA, University of Salerno, Fisciano, Italy; 3 Biogem Scarl, Istituto di Ricerche Genetiche “Gaetano Salvatore” & CaReBios surl, Ariano Irpino, Avellino, Italy; 4 Complex Operating Unit of Thoracic Surgery, Azienda Ospedaliera di Rilievo Nazionale "San Giuseppe Moscati" (A.O.R.N. Giuseppe Moscati), Avellino, Italy

**Keywords:** ceramidase, fibroblasts, hypoxia, metabolic reprogramming, pulmonary fibrosis

## Abstract

**Introduction:**

Physiological metabolic reprogramming is a cells strategy to resist/counteract stress to preserve homeostasis. Hypoxia is an important stress stimulus that can promote tissue remodeling and fibrosis, in which alterations in sphingolipid metabolism may contribute to fibroblast responses. Here, we investigated the potential role of acid ceramidase (ASAH1), a key enzyme of the sphingolipid salvage pathway, in fibroblast adaptation to hypoxic stress.

**Methods:**

Human fibroblasts were exposed to hypoxia for 24, 48, and 72 hours, and metabolic and redox changes were investigated following pharmacological inhibition of acid ceramidase with D-Nmappd. These findings were complemented by analyses of publicly available bulk and single-cell RNA sequencing datasets from idiopathic pulmonary fibrosis (IPF) lungs.

**Results:**

Hypoxic fibroblasts showed metabolic remodeling involving glucose-, amino acid-, and redox-associated pathways, together with changes consistent with an adaptive antioxidant response, including alterations in the GSH/GSSG balance. Pharmacological inhibition of acid ceramidase under hypoxia was associated with changes in carnitine metabolism and long-chain fatty acid β-oxidation, reduced GSH and GSSG levels, and decreased expression of Nuclear Factor Erythroid 2-Related Factor 2 (NRF2), Superoxide dismutase 2 (SOD2), and 8-Oxoguanine DNA Glycosylase 1 (OGG1). In IPF lung tissue, ASAH1 expression was associated with glycolytic and redox-related genes. Single-cell analysis showed that ASAH1-expressing IPF fibroblasts were enriched in transcriptional programs related to hypoxic responses and oxidative stress regulation and displayed features associated with a less activated phenotype.

**Discussion:**

Overall, these findings suggest that acid ceramidase/ASAH1 is associated with fibroblast metabolic and redox adaptation to hypoxic stress and may influence fibroblast activation in IPF.

## Introduction

Cellular metabolic reprogramming is a physiological adaptive response to different types of stimuli, including hypoxia, changes in nutrient availability, inflammation, and infection, allowing cells to maintain homeostasis and preserve functional integrity under stress. Accumulating evidence indicates that the sphingolipids have been implicated in metabolic reprogramming processes across different physio-pathological contexts. In particular, the sphingosine kinase 1 (SPHK1)/sphingosine-1-phosphate (S1P) axis modulates glucose flux and oxygen homeostasis in erythrocytes under hypoxic conditions, as well as in sickle cell disease and chronic kidney disease, by regulating 2,3- bisphosphoglycerate (BPG) production and redox metabolism (Sun et al., 2016; Sun et al., 2017). Sphingolipids have also been shown to drive metabolic adaptations in cancer, including chronic myeloid leukemia, melanoma, and non-small cell lung cancer, where they influence glucose and lipid metabolism, energy production, cell survival, and anti-tumor immune responses (Chakraborty et al., 2019; Xie et al., 2020; Arora et al., 2022; Xie et al., 2025). Collectively, these findings highlight that the physiological adaptation under stress conditions, involves compensatory mechanisms (metabolic shifts, structural remodeling) to preserve functional integrity and to develop strategies to resist or succumb to stress. Hypoxia is a relevant stress stimulus in several pathological conditions and may contribute to fibroblast activation and tissue remodeling, potentially favoring the development and progression of fibrosis. In this context, S1P signaling has been implicated in pro-fibrotic responses, including myofibroblast differentiation and epithelial-to-mesenchymal transition (EMT) (Donati et al., 2021; Osawa et al., 2024). However, whether sphingolipid metabolism, and particularly ceramidase activity, contributes to the physiological metabolic adaptation of fibroblasts during the response to hypoxic stress remains poorly understood. Understanding these adaptive responses may provide insight into mechanisms that precede or contribute to pathological fibroblast activation. Ceramidases are key enzymes of sphingolipid metabolism that hydrolyze ceramide to generate sphingosine, which can subsequently enter the sphingolipid salvage pathway and be phosphorylated to S1P. Through the regulation of ceramide and sphingosine availability, ceramidases may influence cellular signaling, membrane lipid composition, and stress responses, thereby contributing to tissue homeostasis (Kuo and Hla, 2024). Importantly, the biological consequences of ceramidase activity are highly context- and cell-type-dependent, and its contribution to fibroblast adaptation to hypoxic stress remains insufficiently characterized.

Therefore, in the present study, we investigated the potential involvement of acid ceramidase (ASAH1) in the metabolic and redox adaptation of fibroblasts exposed to hypoxic stress. By combining untargeted metabolomics, pharmacological inhibition of acid ceramidase, and analyses of publicly available bulk and single-cell transcriptomic datasets from idiopathic pulmonary fibrosis (IPF) lungs, we aimed to characterize metabolic and transcriptional programs associated with ASAH1 during hypoxic adaptation and to explore their potential relevance to fibroblast activation and fibrotic remodeling.

## Materials and methods

### Hypoxia-induced stress model

Fibroblasts (ATCC^®^ PCS-201-012TM) were cultured in Dulbecco’s Modified Eagle Medium (DMEM) supplemented with 20% Fetal Bovine Serum (FBS), 100 units/ml penicillin, 100 units/ml streptomycin and 2 mM L-glutamine, in an atmosphere of 5% CO_2_ at 37 °C. Cells were seeded (1 × 10^6^ cells/well) and exposed to hypoxia (<1% O_2_) for 24, 48 and 72 hours, in presence or not of a specific inhibitor of ceramidase (D-Nmappd, 5µM) (Raisova et al., 2002; Terlizzi et al., 2021; Terlizzi et al., 2022; Terlizzi et al., 2023), the limiting enzyme of the sphingolipid salvage pathway; hypoxia was induced by incubating cells in a chamber (STEMCELL Technologies) for 4 minutes with a gas flow (5% CO_2_ – 95% N_2_ mixture) at 20 liters per minute. Normoxic fibroblasts were used as controls. The hypoxia chamber was sealed after gas flushing to prevent oxygen diffusion and maintain hypoxic conditions during the experimental exposure.

### MTT assay

To assess cell viability, 3-(4,5-dimethylthiazol-2-yl)-2,5-diphenyltetrazolium bromide (MTT) was added to medium-free cells post 24- and 72-hours treatment. Dimethyl Sulfoxide (DMSO) was used to dissolve the purple formazan crystals. The formazan concentration was determined by measuring the optical density. The data are presented as absorbance (550 nm) versus treatment.

### Bulk RNA sequencing analysis

The public database GSE150910 (https://www.ncbi.nlm.nih.gov/geo/query/acc.cgi?acc=GSE150910) of bulk RNAseq, containing 16680 transcripts, obtained from lung tissues of 103 idiopathic pulmonary fibrosis (IPF) patients, was analyzed in this study. The enrolled subjects were sampled homogeneously considering age, sex, race and smoke history, as reported in the reference paper (Furusawa et al., 2020). A Spearman correlation analysis considering transcripts of interest was performed.

### Single cell RNAseq analysis

The public database GSE136831 (https://www.ncbi.nlm.nih.gov/geo/query/acc.cgi?acc=GSE136831) of scRNAseq, obtained from lung tissues of 28 controls and 32 IPF patients, was analyzed in this study. The enrolled controls subjects were sampled considering age (mean 45.6 years), sex (12 female and 16 male) and smoke history (6 smokers and 22 non-smokers), as reported in the reference paper (Adams et al., 2020); IPF patients’ group was composed of 6 females (age mean 66.8 years; 3 smokers and 3 non-smokers) and 26 males (age mean 65.1 years; 18 smokers and 8 non-smokers). Neighborhood graph construction, dimensionality reduction, and clustering were performed using Scanpy framework. A k-nearest neighbor graph was computed based on the scVI-derived latent representation, capturing cell-to-cell transcriptional similarities in a low-dimensional space. Dimensionality reduction for visualization was carried out using Uniform Manifold Approximation and Projection (UMAP). Graph-based clustering was then performed using Leiden algorithm to identify transcriptionally distinct cell populations, as already reported (Terlizzi et al., 2024). Cell phenotype annotation was performed by examining the expression profiles of known marker genes for each cell type. Subsequently, sub-clustering of stromal compartment was performed following the same procedure, allowing for higher-resolution identification of cellular populations of interest, in particular fibroblasts.

### Metabolite extraction from fibroblasts

The preparation of the samples (n = 5 for each experimental group) intended for untargeted metabolomic analysis was as follows. Each of the five samples represented an independent biological replicate obtained from a separate cell culture and processed independently throughout the entire experimental workflow. Equal numbers of fibroblasts (1 × 10^6^ cells/well) were seeded and maintained under identical experimental conditions prior to metabolite extraction. After the experimental time (24, 48 and 72 hours), the supernatant was removed and the cells (fibroblasts) were rinsed with ice-cold 0.9% saline twice; saline was aspirated and 500 µL/well of cell extraction solution (MeOH/H2O 80/20 v/v kept in -80 °C) were added; the cells were scraped, transferred into a pre-chilled tube and stored at -80 °C until the experiments were performed, when cell pellets were thawed on ice, and 300 μL of ice-cold MeOH 80% (v/v) containing a mixture of deuterated standards were added and sonicated for 10 minutes. Subsequently, the solution was continuously agitated in a thermomixer for 10 minutes, 300 rpm at 4 °C. Then, samples were incubated at -30 °C for 30 minutes and centrifuged at 14680 rpm for 10 minutes at 4 °C to induce phase separation. The supernatants were collected, evaporated using a vacuum concentrator and then solubilized in 80 μL of acetonitrile (CAN) 70% (v/v) before injection in Ultra-High-Performance Liquid Chromatography-High Resolution Mass Spectrometry (UHPLC-HRMS).

### Instrumentation and LC setup

Pooled quality control (QC) samples were prepared by combining equal aliquots from all study samples and were analyzed periodically throughout the analytical sequence to monitor instrument stability and analytical reproducibility. Metabolome analyses were performed on a Thermo Vanquish Flex UHPLC coupled online to hybrid quadrupole Orbitrap Exploris 120 mass spectrometer (Thermo Fisher Scientific, Bremen, Germany) equipped with a heated electrospray ionization probe (HESI II). Cell-culture derived metabolites were separated using an Acquity UPLC BEH Amide™ (100 × 2.1 mm × 1.7 µm, 130 Å) protected with a VanGuard BEH Amide™ precolumn (5.0 × 2.1 mm; 1.7 µm, 130 Å) (Waters, Milford, MA, USA). The mobile phases were composed of (A): H_2_O/ACN (95:5 v/v%) and (B): H_2_O/ACN (5:95 v/v%), both with 0.1% HCOOH (v/v %) + 10 mM HCOONH_4_ were employed for positive and negative analyses. The following gradient was used: 0–1 min, 99%B, 7–8 min 30% B, 8.1-11.5 min 99% B. The column temperature was set at 45 °C and a flow rate of 0.4 mL/min was used. The injection volume was 2 µL.

### HRMS parameters

MS data acquisition was performed in full scan-data dependent acquisition (FS-DDA) in the m/z range 70–800. MS1 scan OT resolution, 60,000; AGC, auto; maximum injection time, 100 ms. S-Lens RF level, 70; ddMS2 OT resolution, 15,000; isolation window, 1.5 Da; dynamic exclusion, 10 s; AGC, auto; maximum injection time, 22 ms. TopN, 4; HCD fragmentation normalized collision energies (NCE): 20, 40, 60. The HESI source parameters were as follows: sheath gas pressure, 40 a.u. and 50 a.u. for positive and negative ionization modes, respectively; aux gas flow, 15 a.u.; sweep gas flow, 0 a.u. Spray voltages were set to 3.3 kV and 3.0 kV for ESI (+) and ESI (−). Ion transfer tube (ITT) and vaporizer temperatures were set to 300 °C and 320 °C. The same MS and MS/MS acquisition parameters and calibration were used as reported above. Data were kindly performed by technicians, collaborators at the Department of Pharmacy, University of Salerno.

### Data processing and statistical analysis

All samples were analyzed in a single analytical batch; therefore, no batch-effect correction was required prior to statistical analysis. FreeStyle (Thermo Fisher Scientific) was used to visualize RAW data, which were then imported to Compound Discoverer v.3.3 (Thermo Fisher Scientific) to normalize, align, detect, and identify compounds. Features were extracted from 0–10 min and 0–11 min of the HILIC and RP chromatography runs, respectively, in the m/z = 70–800 mass range. Data were aligned according to an adaptive curve alignment model. Compounds were detected using the following parameters settings: mass tolerance was set to 5 ppm, while retention time tolerance was set to 0.2 min; minimum peak intensity was set to 100,000 AU and the signal to noise threshold for compound detection was set to 5. The peak rating filter was set to 3. To perform blank subtraction, we maintained max sample/max blank ratio > 5. For statistical comparison between the two setups, signal intensity was normalized by using the algorithm “Constant Sum.” To predict elemental compositions of the compounds, the relative intensity tolerance was set to 30% for isotope pattern matching. For the mzCloud database search, both the precursor and fragment mass tolerance were set to 5 ppm. The databases used for matching compounds in ChemSpider for structural search were BioCyc, the Human Metabolome Database, and KEGG, and the mass tolerance in ChemSpider Search was set to 5 ppm. The mass tolerance for matching compounds in Metabolika pathways was set to 5 ppm. Compounds were assigned by comparing annotations using the following nodes in order of priority: (1) mzCloud; (2) Predicted Compositions; (3) MassList search; (4) ChemSpider Search; (5) Metabolika search. Metabolite annotations were assigned according to the Metabolomics Standards Initiative criteria as putatively identified compounds (MSI level 2), based on accurate mass and MS/MS spectral matching against the mzCloud spectral library.

### Metabolite set enrichment analysis

By using MetaboAnalyst 6.0 web-based platform, HILIC-HRMS data were normalized, a direct comparison of the mean abundance of metabolites between two experimental conditions (e.g., hypoxia vs. normoxia) was performed, and the fold change (FC) and Log2FC values were obtained. Metabolites showing a FC > 1 were selected for exploratory pathway enrichment analysis using Over-Representation Analysis (ORA). This analysis was intended to identify metabolic pathways potentially associated with the observed changes and was therefore considered hypothesis-generating. The one-tailed hypergeometric test was used to calculate the probability of observing a certain number of significant metabolites within the chosen reference metabolite library (Small Molecule Pathway Database, SMPDB). p-values were corrected for multiple testing using the Benjamini–Hochberg False Discovery Rate (FDR) method.

### Flow cytometry analysis

Fibroblasts were exposed to hypoxia for 72 hours, in presence or not of D-Nmappd 5µM; normoxic fibroblasts were used as controls. Independent biological replicates were used. The percentage of human fibroblasts positive to NRF2 (Nuclear Factor Erythroid 2-Related Factor 2; Santa Cruz Biotechnology, #sc-365949), SOD2 (Superoxide dismutase 2; Santa Cruz Biotechnology, #sc-30080), OGG1 (8-Oxoguanine DNA Glycosylase 1; Origene, #TA321496), FAP (Fibroblast Activation Protein; Proteintech, #84018-4-PR), αSMA (Alpha Smooth Muscle Actin; Elabscience, #E-AB-34268) and SPNS2 (Spinster homolog 2; Abcam, #Ab82629) was analyzed by using flow cytometry (BD FacsCalibur, Milan, Italy). In another set of experiments, fibroblasts were stained for TetraMethyl Rhodamine Esther (TMRE, 5 nM, Life Technologies, Monza, Italy) to measure mitochondrial membrane potential alterations, and for MitoSOX Mitochondrial Superoxide Indicator as indicated in the manufacturer’s guide (Life Technologies, USA). Flow cytometry data were analyzed after exclusion of debris based on forward and side scatter parameters, and fluorescence positivity was determined using unstained controls.

### STRING analysis

To characterize the fibrotic fibroblasts molecular functions compared to controls, a functional enrichment analysis was performed by means of STRING platform (version 12.0; https://string-db.org/). A public single cell (sc) RNAseq dataset (GSE136831) of lung tissues from controls and IPF patients was analyzed, and 376 differentially expressed transcripts (DETs) significantly up-regulated in IPF compared to controls were considered and subjected to STRING analysis; the following setting has been set for the analysis: network type: full STRING network; meaning of network edges: confidence; active interaction source: textmining, experiment, databases, co-expression; minimum required interaction score: highest confidence 0.9.

### Pathway enrichment analysis

To characterize the transcriptional programs associated with acid ceramidase (ASAH1) expression in IPF fibroblasts from scRNAseq dataset (GSE136831), single-cell transcript counts from the fibroblasts cluster were aggregated at the patient level to generate pseudobulk expression profiles. Differential gene expression analysis was performed using the PyDESeq2 implementation of DESeq2, and IPF patients were stratified into ASAH1+ and ASAH1- groups according to the median normalized ASAH1 expression across the cohort. Differentially expressed genes (DEGs) were then identified by comparing ASAH1+ versus ASAH1- patients within the DESeq2 framework. Genes were subsequently ranked according to the Wald statistic and subjected to Gene Set Enrichment Analysis (GSEA) using GSEApy. Enrichment analysis was performed against the GO Biological Process, GO Molecular Function, and Reactome Pathways gene set collections using 1,000 permutations. Pathways with a false discovery rate (FDR) < 0.05 were considered significantly enriched.

### Statistical analysis

Data are reported as median and represented as scatter dot plots. Statistical differences were assessed with Kruskal Wallis test followed by Dunn’s *post-hoc* test. p values less than 0.05 were considered significant. The statistical analysis was performed by using GraphPad prism 10.1.0 version (San Diego, CA, USA).

## Results

### Hypoxia-induced stress stimulus reprogrammed fibroblasts toward an anti-oxidant profile

In order to understand whether a stimulus of hypoxia could reprogram fibroblast metabolism, we subjected cells to hypoxia and the metabolomic profile was evaluated 24, 48 and 72 hours following the hypoxic stimulus. As control group, cells were exposed to the same incubator air with normal conditions of O_2_ and CO_2_.

Exploratory pathway enrichment analysis of the metabolomic data identified metabolic pathways potentially associated with the response of fibroblasts to hypoxia, including the glucose-alanine cycle, glycine-serine cycle, glutamate, purine, histidine and methylhistidine, β-alanine metabolism, associated to the Warburg effect (**Figure 1A**; blue arrows; **Supplementary Table 1**; **Supplementary Figure 1A**). These pathway-level findings are consistent with metabolic remodeling of fibroblasts in response to hypoxic stress. To avoid any technical bias, the viability of cells was also checked and no alteration was observed at this experimental condition (**Supplementary Figure 1B**). This metabolomic profile was visible from 24 (**Figure 1A**) up to 48 (**Figure 1B**; **Supplementary Table 2**) and 72 hours (**Figure 1C**; **Supplementary Table 3**).

**Figure 1:**
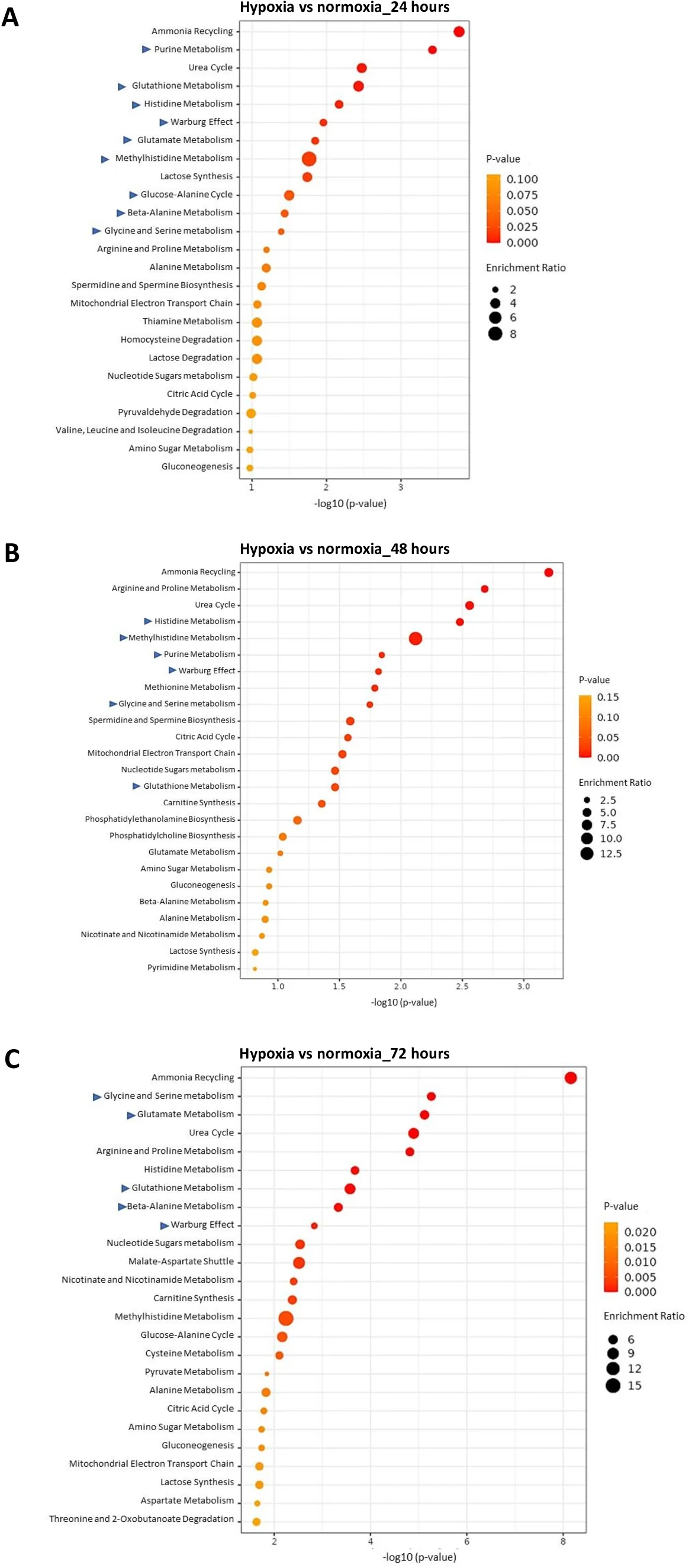
Enriched (top 25) metabolic pathways in hypoxia- compared to normoxia-exposed fibroblasts. Human fibroblasts were exposed to hypoxia for 24 **(A)**, 48 **(B)** and 72 **(C)** hours and an untargeted metabolomic analysis was performed; resulting data were analyzed with MetaboAnalyst 6.0 tool and a metabolite set enrichment analysis (MSEA) was carried out. Dot plot of the enrichment analysis results of hypoxic compared to normoxic fibroblasts. The size of the circles per metabolite set represents the enrichment ratio and the color represents the p value.

Consistent with a potential adaptive metabolic response, hypoxic fibroblasts showed increased abundance of metabolites associated with antioxidant activity (Yang and Vousden, 2016; Vera-Aviles et al., 2018; Aldini et al., 2021; Franco et al., 2022; Pérez de la Lastra et al., 2023; Liu et al., 2025; Movahed et al., 2026), including carnosine (**Figure 2A**) and metabolites indirectly involved in antioxidant metabolism, such as L-glutamine, L-histidine, L-serine, ornithine, pantothenic acid, 4-pyridoxic acid and homo-L-arginine (**Figures 2B–H**), compared with normoxic fibroblasts. In addition, hypoxic fibroblasts showed a shift in the GSH/GSSG balance toward the reduced form (**Supplementary Figure 1C**), providing metabolite-level evidence consistent with maintenance of redox homeostasis. These data imply that a stimulus like hypoxia reprogram fibroblasts in their metabolism and ensures redox homeostasis.

**Figure 2:**
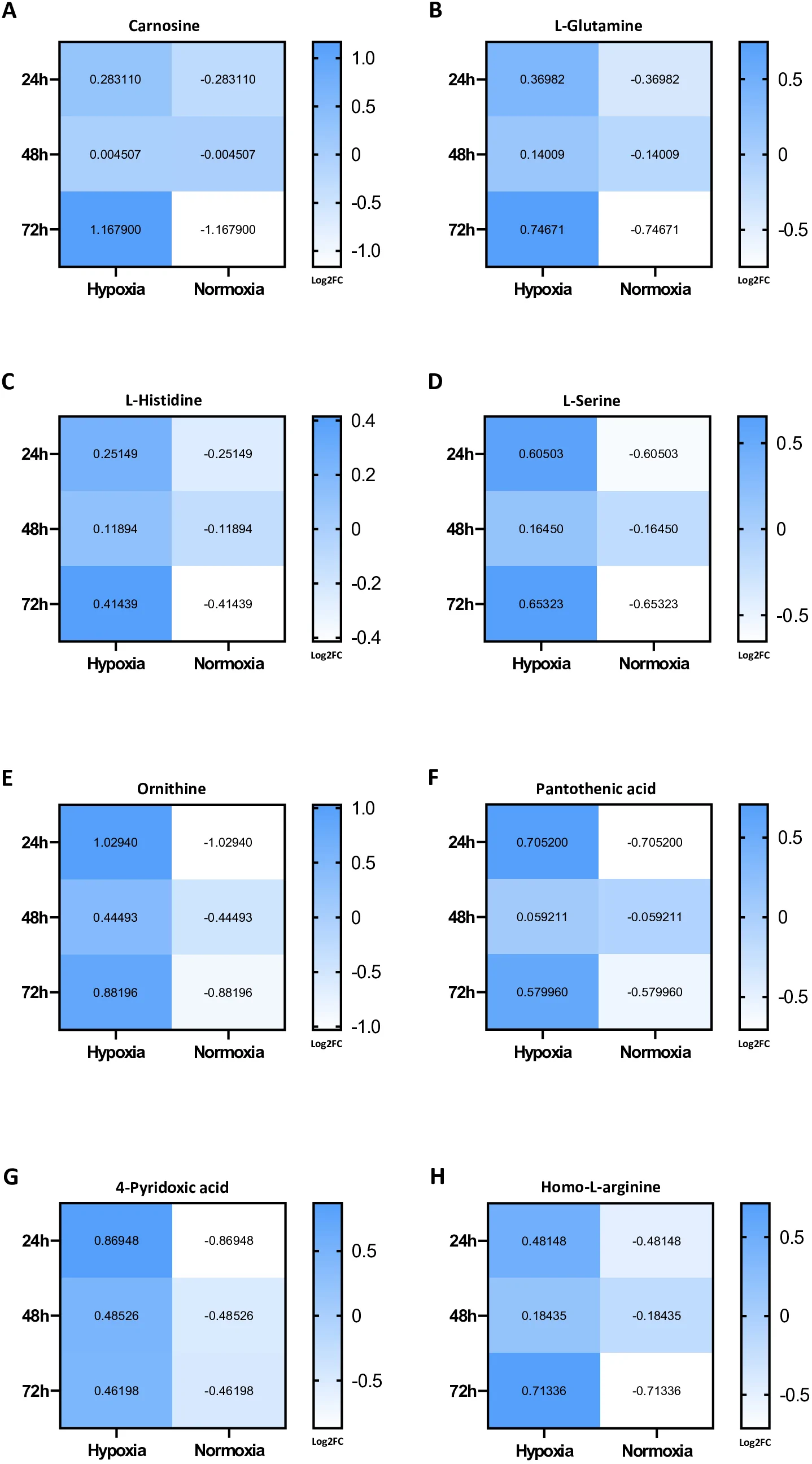
Anti-oxidant metabolites in hypoxia- compared to normoxia-exposed fibroblasts. Human fibroblasts were exposed to hypoxia for 24, 48 and 72 hours and an untargeted metabolomic analysis was performed; resulting data were analyzed with MetaboAnalyst 6.0 tool and the abundance of metabolites between two experimental conditions (hypoxia vs. normoxia) was evaluated. **(A)** Carnosine; **(B)** L-glutamine; **(C)** L-histidine; **(D)** L-serine; **(E)** Ornithine; **(F)** Pantothenic acid; **(G)** 4-pyridoxic acid; **(H)** Homo-L-arginine. Data are expressed as Log2 Fold Change (FC).

### Pharmacological inhibition of acid ceramidase is associated with metabolic remodeling in hypoxic fibroblasts

Recent studies show that metabolic adaptation to hypoxia can be regulated by intracellular sphingolipid-associated pathways (Xie et al., 2020; Hay et al., 2023). To understand the role of sphingolipids in the metabolic switch necessary for fibroblast adaptation after a hypoxic stimulus, we used a specific inhibitor of the ceramidase (D-Nmappd, 5µM), a limiting enzyme of the sphingolipid salvage pathway. Viability of cells was not altered (**Supplementary Figure 1D**), and a metabolomic analysis was performed (**Figure 3**).

**Figure 3:**
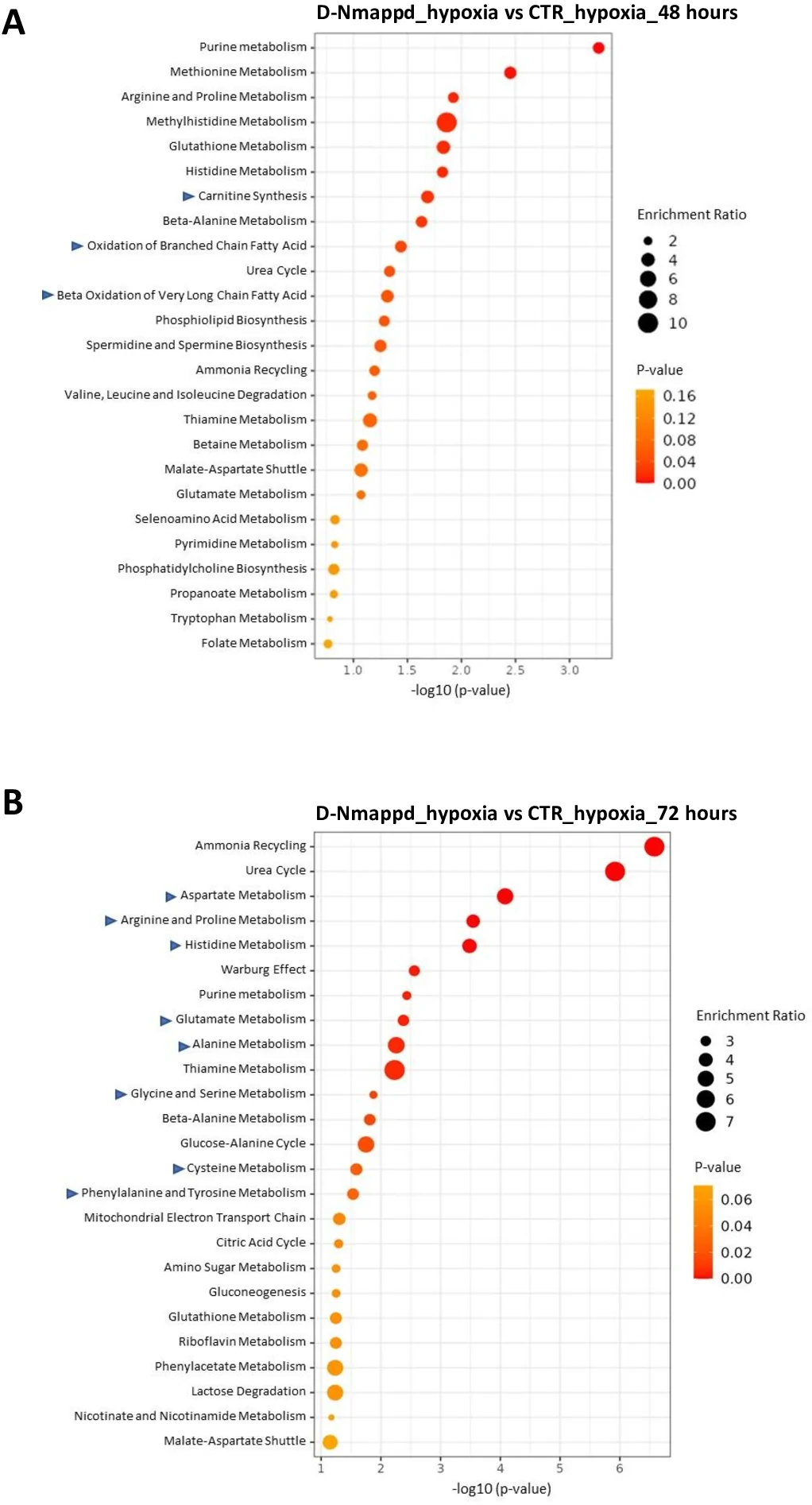
Enriched (top 25) metabolic pathways in D-Nmappd_hypoxia- compared to CTR_hypoxia-exposed fibroblasts. Human fibroblasts were exposed to hypoxia for 48 **(A)** and 72 **(B)** hours in presence of a specific inhibitor of ceramidase (D-Nmappd 5µM) and an untargeted metabolomic analysis was performed; resulting data were analyzed with MetaboAnalyst 6.0 tool and a metabolite set enrichment analysis (MSEA) was carried out. Dot plot of the enrichment analysis results of D-Nmappd_hypoxic compared to CTR_hypoxic fibroblasts. The size of the circles per metabolite set represents the Enrichment Ratio and the color represents the p value.

Exploratory pathway enrichment analysis of D-Nmappd-treated hypoxic fibroblasts identified pathways potentially related to carnitine metabolism and long-chain fatty acid β-oxidation at 48 hours (**Figure 3A**, blue arrows; **Supplementary Table 4**), whereas amino acid metabolism-related pathways emerged at 72 hours (**Figure 3B**, blue arrows; **Supplementary Table 5**). These findings suggest that pharmacological perturbation of acid ceramidase may be associated with remodeling of alternative metabolic pathways under hypoxic stress. To note, at 24 hours, no pathway reached the predefined pathway-level significance threshold following D-Nmappd treatment (**Supplementary Figure 1E**), suggesting that the metabolic changes associated with pharmacological acid ceramidase perturbation may become more evident at later time points.

Consistent with the metabolic changes identified by the exploratory pathway analysis, inhibition of acid ceramidase was associated with reduced levels of both GSH and GSSG (**Supplementary Figures 1F, G**). In particular, GSH and GSSG were down-regulated when ceramidase was inhibited compared to hypoxic control, with a delta of 0.734 at 24 hours and 1.192 at 72 hours for GSH (**Supplementary Figure 1F**), and of 2.098 at 24 hours and of 0.045 at 72 hours for GSSG (**Supplementary Figure 1G**). To confirm these metabolomic data, the dysregulation of anti-oxidant mechanism in D-Nmappd-treated hypoxic cells was also evaluated by flow cytometry analysis. The inhibition of ceramidase with D-Nmappd reduced the expression of anti-oxidant enzymes, such as Nuclear Factor Erythroid 2-Related Factor 2 (NRF2, **Figure 4A**) and Superoxide dismutase 2 (SOD2, **Figure 4B**), and of enzymes involved in the DNA repair, such as 8-Oxoguanine DNA Glycosylase 1 (OGG1, **Figure 4C**). These findings suggest that pharmacological inhibition of acid ceramidase is associated with alterations in antioxidant and DNA-repair responses during hypoxic stress.

**Figure 4:**
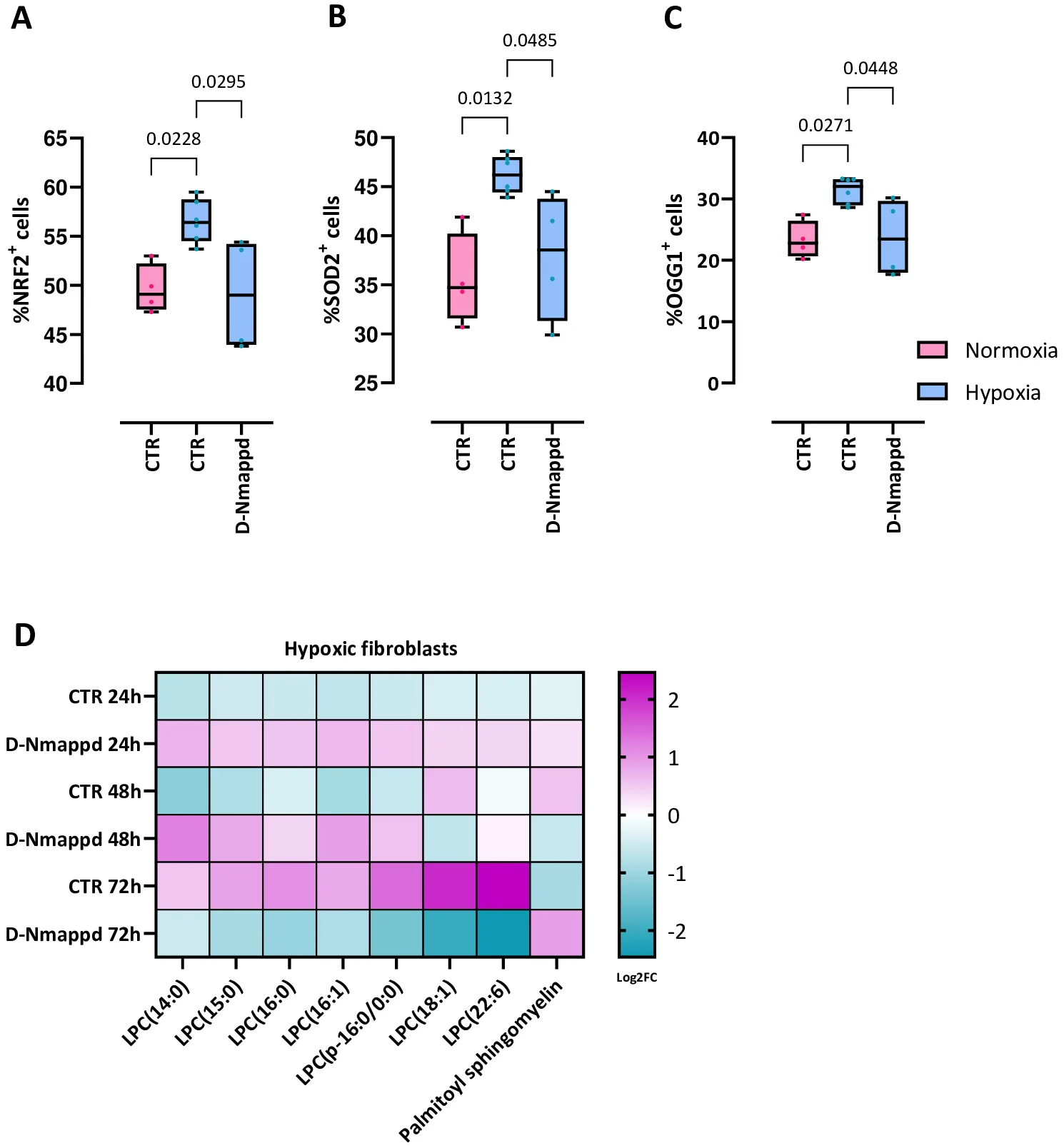
Human fibroblasts were exposed to hypoxia for 72 hours, in presence of ceramidase inhibitor (D-Nmappd, 5 µM); the percentage of cells positive to NRF2 **(A)**, SOD2 **(B)**, OGG1 **(C)**, was evaluated by flow cytometry analysis, in normoxic (light red box) and hypoxic (light blue box) condition. Data are reported as median and represented as box plots with individual data points. Statistical differences between medians were assessed through Kruskal Wallis test. Each dot represents an independent biological replicate. NRF2: Nuclear Factor Erythroid 2-Related Factor 2; SOD2: Superoxide dismutase 2; OGG1: 8-Oxoguanine DNA Glycosylase 1. **(D)** Heatmap representing the abundance of metabolites between CTR_hypoxic and D-Nmappd_hypoxic fibroblasts at 24, 48 and 72 hours, after untargeted metabolomic analysis. Data are expressed as Log2 Fold Change (FC) of metabolite in CTR_hypoxic vs D-Nmappd_hypoxic group and vice versa. LPC, lysophosphatidylcholine.

In support, hypoxic fibroblasts exposed for 24 and 48 hours to D-Nmappd showed an enrichment of lysophospholipids, i.e. lysophosphatidylcholine (LPC) 14:0, 15:0, 16:0, 16:1, p-16:0/0:0 and 22:6 compared to hypoxic control (**Figure 4D**), balanced by an increase of palmitoyl sphingomyelin after 72 hours (**Figure 4D**).

To further investigate the metabolic consequences of ceramidase inhibition under hypoxic conditions, mitochondrial function was assessed. D-Nmappd-treated hypoxic fibroblasts showed a trend toward an increased percentage of TMRE-positive cells, suggesting preservation of mitochondrial membrane potential (**Supplementary Figure 2A**). At the same time, these cells exhibited a significant increase in Mitosox-positive cells, indicating enhanced mitochondrial superoxide production (**Supplementary Figure 2B**). Consistent with these findings, intracellular lactic acid levels were significantly reduced compared with hypoxic control cells (**Supplementary Figure 2C**).

Collectively, these data support a reduced reliance on glycolytic metabolism and a relative shift toward mitochondrial oxidative metabolism following ceramidase inhibition under hypoxic adaptation.

### Ceramidase expression is associated with metabolic transcriptional signatures in IPF lungs: bulk RNAseq analysis

Since hypoxia is a sentinel feature of idiopathic pulmonary fibrosis (IPF), in the development and progression of which fibroblasts play a decisive role (Yang et al., 2023; Lebel et al., 2024), we moved on by performing a transcriptomic analysis based on public bulk RNAseq dataset from IPF patients.

Transcriptomic profiles from 103 IPF lung samples (GSE150910) were analyzed to explore associations between ASAH1 (ceramidase, N-acylsphingosine amidohydrolase 1) expression and genes involved in metabolic and redox-related pathways. At the whole-lung transcriptomic level, ASAH1 expression was positively correlated with pyruvate dehydrogenase kinase (PDK3, r=0.4607, p<0.0001; **Figure 5A**) and lactate dehydrogenase B (LDHB, r=0.4973, p<0.0001; **Figure 5B**), two genes associated with glycolytic metabolism and Warburg effect, and was negatively correlated to carnitine palmitoyltransferase 1C (CPT1C, r=-0.5329, p<0.0001; **Figure 5C**), a gene involved in fatty acid β-oxidation.

**Figure 5:**
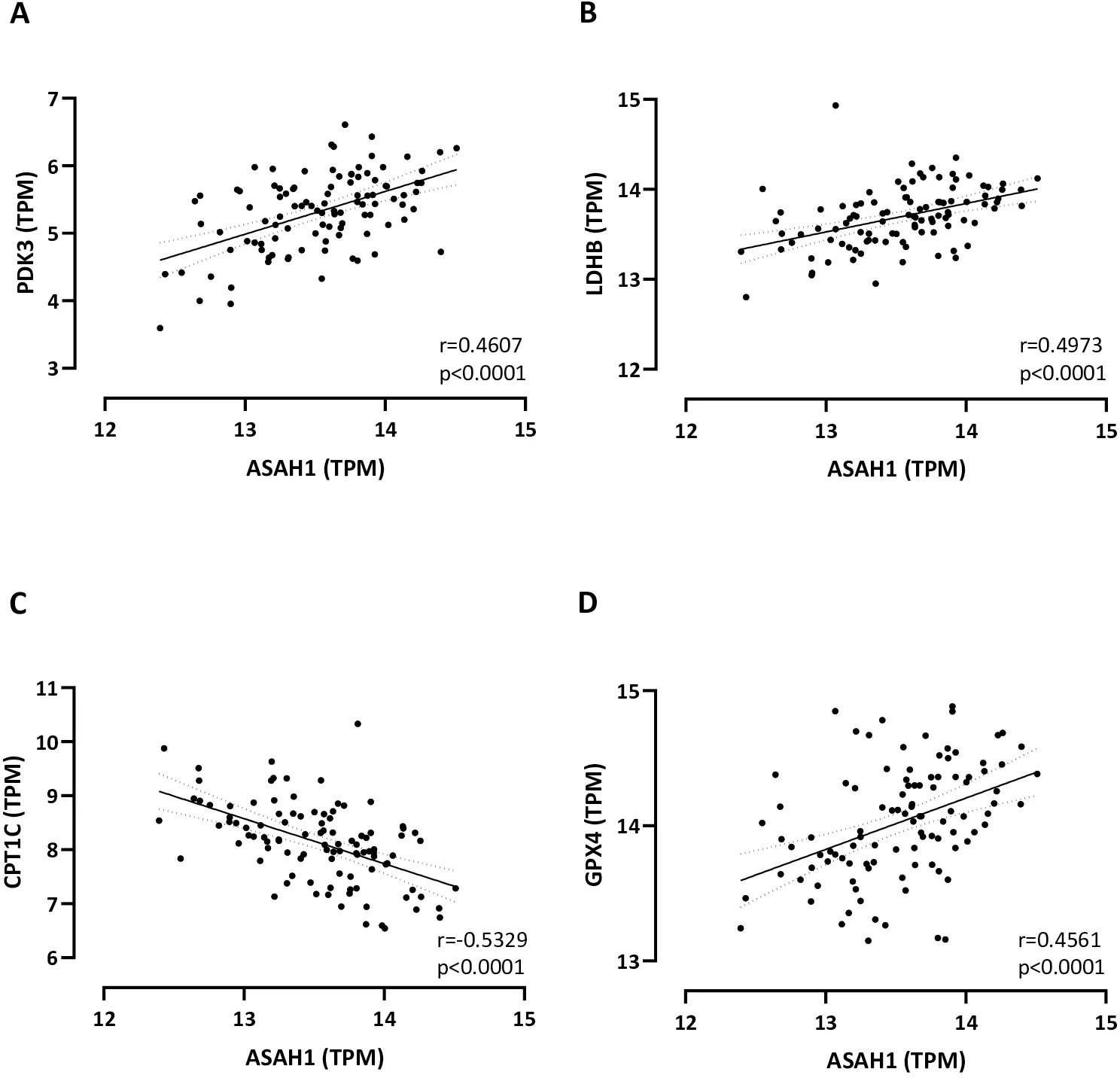
Transcriptomic analysis of bulk RNAseq dataset (GSE150910) from 103 lung samples (whole lung tissues) of idiopathic pulmonary fibrosis (IPF) patients, revealed that ceramidase (ASAH1) was positively correlated to PDK3 [**(A)** Spearman r = 0.4607, 95% CI = 0.2881 to 0.6044; p<0.0001], LDHB [**(B)** Spearman r = 0.4973, 95% CI = 0.3310 to 0.6337; p<0.0001] and GPX4 [**(D)** Spearman r = 0.4561, 95% CI = 0.2827 to 0.6007; p<0.0001], and negatively correlated to CPT1C [**(C)** Spearman r = -0.5329, 95% CI = -0.6618 to -0.3735; p<0.0001]. Data represent a nonparametric Spearman correlation. ASAH1, N-acylsphingosine amidohydrolase 1; PDK3, pyruvate dehydrogenase kinase; LDHB, lactate dehydrogenase B; CPT1C, carnitine Palmitoyltransferase 1C; GPX4, glutathione peroxidase 4.

In addition, ASAH1 expression was positively correlated with glutathione peroxidase 4 (GPX4, r=0.4561, p<0.0001; **Figure 5D**), one of the most important enzymes involved in cellular protection against oxidative stress, especially that affecting membrane lipids.

Overall, these whole-lung transcriptomic associations indicate a relationship between ASAH1 expression and glycolytic and redox-related transcriptional programs in IPF lung tissue.

### Ceramidase expression is associated with transcriptional features related to redox homeostasis in IPF fibroblasts: transcriptomic analysis of scRNAseq data

To provide complementary cell-type context for the associations observed in the bulk RNA-seq analysis, and to verify which cell population was primarily involved in the ceramidase-dependent metabolic adaptation induced by hypoxia, we took advantage of public single cell (sc) RNAseq dataset (GSE136831) of lung tissues from controls and IPF patients. Clustering analysis was performed (**Supplementary Figure 2D**) and the expression of ceramidase (ASAH1) was evaluated. We found that fibroblasts of IPF patients showed an over-transcription of ASAH1 compared to controls (**Figure 6A**), and compared to all other cell populations (**Figure 6B**, red arrow). To characterize the lung fibroblasts of IPF patients’ molecular functions compared to controls, a functional enrichment analysis was performed by means of STRING platform; specifically, 376 differentially expressed transcripts (DETs) significantly up-regulated in IPF compared to controls were considered. As expected, we found that fibroblasts from IPF patients performed a normal physiological function, in that pathways associated to extracellular matrix organization/remodeling were enriched, compared to controls (**Figure 6C**).

**Figure 6:**
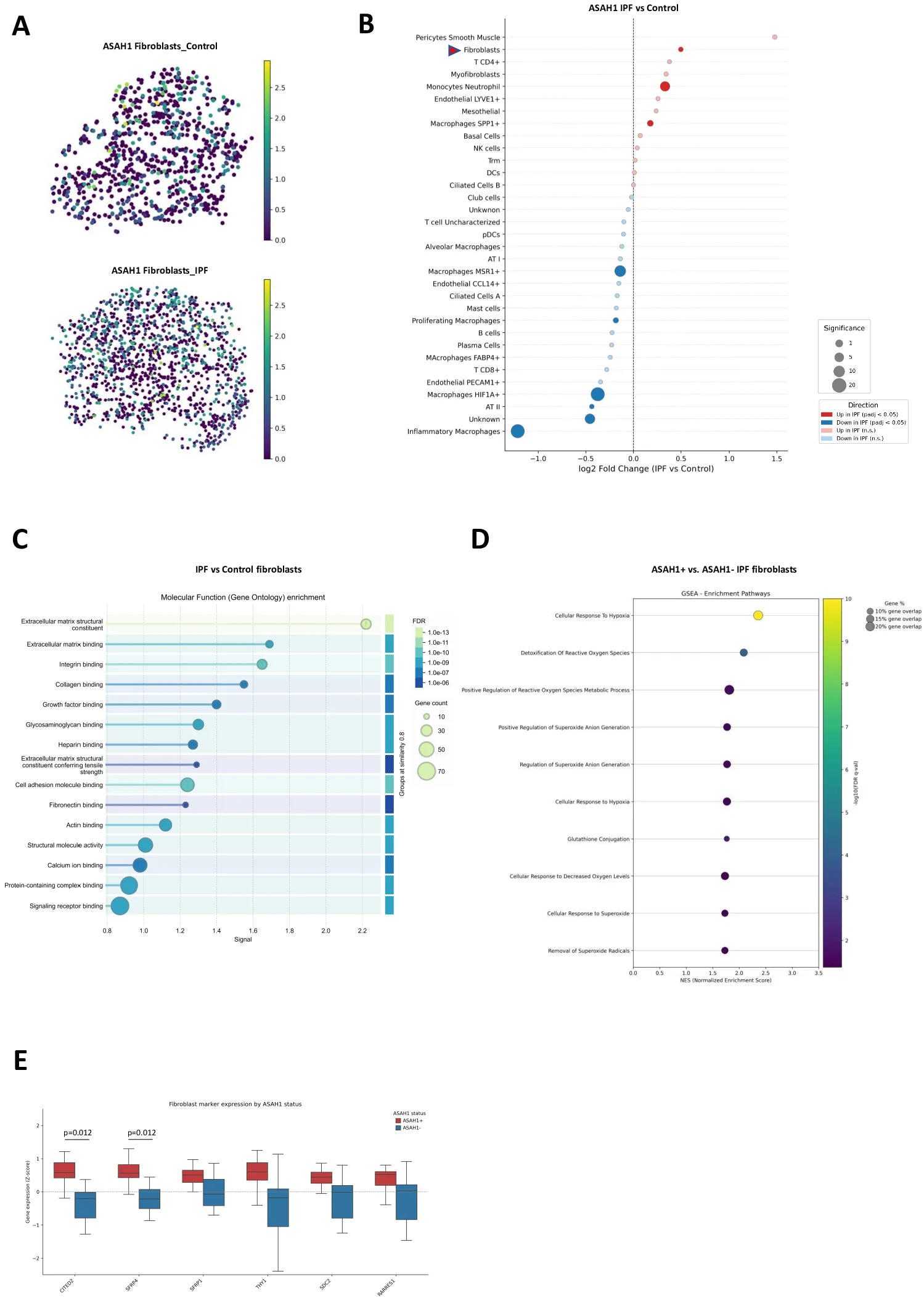
Transcriptomic analysis of single cell (sc) RNAseq dataset (GSE136831) from 28 controls and 32 idiopathic pulmonary fibrosis (IPF) patients (lung tissues). Ceramidase (ASAH1) expression in fibroblasts cluster in control and IPF patients **(A)**, and in all cell populations of the dataset **(B)**; data are expressed as Log2 Fold Change (FC) of transcript in IPF compared to controls. The size of the circles per cell population represents the significance, while the color represents the enrichment direction. **C)** STRING analysis on 376 differentially expressed transcripts (DETs) significantly up-regulated in fibroblasts of IPF patients compared to controls, shows the enriched pathways in IPF fibroblasts; top 15 Molecular Function of Gene Ontology (GO) enriched pathways are represented. **(D)** Gene Set Enrichment Analysis (GSEA) analysis between ASAH1+ vs ASAH1- fibroblasts from IPF patients. Dot size represents the percentage of gene overlap within each gene set, whereas color indicates the adjusted p value. **(E)** Expression of negative (CITED2, SFRP4, SFRP1, THY1, SDC2, and RARRES1) modulators of fibroblasts to myofibroblasts transition (FMT) in ASAH1+ compared to ASAH1- fibroblasts. Data are expressed as standardized expression of transcripts in the groups. CITED2 (Cbp/P300 Interacting Transactivator With Glu/Asp Rich Carboxy-Terminal Domain 2), SFRP4 (Secreted Frizzled Related Protein 4), SFRP1 (Secreted Frizzled Related Protein 1), THY (Thy-1 Cell Surface Antigen), SDC2 (Syndecan 2), RARRES1 (Retinoic Acid Receptor Responder 1).

To further explore the transcriptional features associated with ASAH1 expression in fibroblasts, and considering that ASAH1 was expressed in 35.9% of IPF fibroblasts (**Supplementary Figure 2E**), compared to 27% of control fibroblasts, a pseudobulk analysis on fibroblasts cluster was performed by aggregating fibroblast gene expression for each patient. Patients were then stratified into ASAH1+ and ASAH1- groups based on the median normalized ASAH1 expression. Differential gene expression analysis was performed between the two groups, and the resulting ranked gene list was subjected to Gene Set Enrichment Analysis (GSEA). This analysis revealed that ASAH1+ IPF fibroblasts were enriched for transcriptional programs related to the hypoxic response and the regulation of oxidative stress (**Figure 6D**).

Altogether, these data indicate that ASAH1 expression in IPF fibroblasts is associated with transcriptional programs related to hypoxic adaptation and oxidative stress regulation.

### Ceramidase expression is associated with a transcriptional feature consistent with a less activated fibroblasts phenotype in IPF

To further characterize the transcriptional features associated with ASAH1 expression in IPF fibroblasts, particularly in relation to fibroblast activation, we analyzed the expression of transcripts implicated in the regulation of fibroblast-to-myofibroblast transition (FMT); a differential gene expression analysis between ASAH1+ and ASAH1- IPF fibroblasts was performed. We found that the negative regulators of FMT, CITED2 (Cbp/P300 Interacting Transactivator With Glu/Asp Rich Carboxy-Terminal Domain 2) and SFRP4 (Secreted Frizzled Related Protein 4) were significantly up-regulated in ASAH1+ compared to ASAH1- patients; in addition, a trend toward higher expression was observed for other reported negative modulators of FMT, including SFRP1 (Secreted Frizzled Related Protein 1), THY (Thy-1 Cell Surface Antigen), SDC2 (Syndecan 2) and RARRES1 (Retinoic Acid Receptor Responder 1) (**Figure 6E**). These associations suggest a transcriptional profile compatible with reduced fibroblast activation in ASAH1+ compared with ASAH1- IPF fibroblasts; however, they do not by themselves establish a functional inhibition of FMT.

To functionally validate these transcriptional findings, the expression of α-SMA (Alpha Smooth Muscle Actin), a well-known marker of myofibroblasts differentiation (Tai et al., 2021), was evaluated by flow cytometry in hypoxic fibroblasts following pharmacological inhibition of ceramidase. As expected, hypoxia increased α-SMA expression compared with normoxic control fibroblasts (**Supplementary Figure 2F**). Importantly, treatment with D-Nmappd further increased α-SMA expression compared with hypoxic control cells (**Supplementary Figure 2F**), indicating that ceramidase inhibition was associated with an increased myofibroblast-like phenotype under hypoxic conditions. These complementary transcriptional and functional data support the hypothesis that ASAH1 expression is associated with a less activated fibroblast transcriptional phenotype, while alterations in ceramidase activity may influence fibroblast activation under hypoxic conditions.

## Discussion

In the present study, we combined untargeted metabolomics with transcriptomic analyses of public bulk and scRNA sequencing datasets to investigate the potential involvement of acid ceramidase (ASAH1) in the metabolic adaptation of fibroblasts exposed to hypoxic stress and in the context of idiopathic pulmonary fibrosis (IPF). Overall, our findings indicate that ASAH1 expression/activity is associated with metabolic programs involved in the adaptation of fibroblasts to hypoxic stress and with transcriptional signatures consistent with a less activated fibroblast phenotype in fibrotic lungs.

Acid ceramidase is a key enzyme of sphingolipid metabolism and contributes to the sphingolipid salvage pathway by hydrolyzing ceramide to sphingosine, thereby influencing the balance among ceramide, sphingosine and downstream sphingolipid metabolites. Its biological effects are highly dependent on the cellular and pathological context. Indeed, acid ceramidase has been implicated in several immune and stromal cell functions, including inflammatory responses, cell survival, differentiation and tissue remodeling (Duarte et al., 2020; Thakkar et al., 2025). Thus, rather than exerting a uniform biological effect, acid ceramidase activity may contribute to distinct cellular responses depending on the metabolic and signaling environment. In this context, its contribution to fibroblast adaptation to hypoxic stress remains poorly characterized.

Our metabolomic analysis revealed substantial metabolic differences between hypoxic fibroblasts and hypoxic fibroblasts treated with D-Nmappd. In untreated hypoxic fibroblasts, enrichment of pathways related to glycolytic adaptation, redox homeostasis and antioxidant metabolism was observed. These findings are consistent with the ability of fibroblasts to undergo metabolic remodeling in response to oxygen deprivation while maintaining cellular viability and redox balance. In contrast, pharmacological inhibition of acid ceramidase was associated with enrichment of pathways involved in carnitine metabolism and long-chain fatty acid β-oxidation. These observations suggest that perturbation of acid ceramidase activity may modify the metabolic strategies adopted by fibroblasts under hypoxic stress, potentially favoring alternative energy-generating pathways.

In parallel, pharmacological inhibition of acid ceramidase was accompanied by metabolic changes involving membrane lipid remodeling, including alterations in lysophosphatidylcholine and sphingomyelin species, which are compatible with cellular adaptation to metabolic and oxidative stress. These metabolic observations were complemented by transcriptomic analysis of a public bulk RNAseq dataset (GSE150910) from lung tissues of IPF patients, which showed that ASAH1 expression was positively correlated with pyruvate dehydrogenase kinase (PDK3) and lactate dehydrogenase B (LDHB), two markers of the glycolytic metabolism and Warburg effect, crucial to prevent pyruvate usage by mitochondria to be fully oxidized and converted to lactate, and was negatively correlated to carnitine palmitoyl transferase 1C (CPT1C), marker of fatty acid β-oxidation. In addition, ASAH1 was also positively correlated with glutathione peroxidase 4 (GPX4), one of the most important enzymes involved in cellular protection against oxidative stress, especially that affecting membrane lipids. GPX4 reduces peroxidized lipids (L-OOH) to non-toxic lipid alcohols (L-OH) using glutathione (GSH); this prevents damage to the cell membrane and its associated proteins. Indeed, a reduction in GSH, as observed in D-Nmappd-treated hypoxic fibroblasts compared with hypoxic controls, may be associated with impaired GPX4 function and increased oxidative stress, whereas the increased GSH observed in hypoxic controls compared with normoxic controls may contribute to the maintenance of redox homeostasis (Zhang et al., 2024). Nevertheless, direct measurements of ASAH1 enzymatic activity and GPX4 activity would be required to establish a mechanistic link between these pathways. Together, these findings identify a transcriptional signature linking ASAH1 expression with metabolic pathways involved in hypoxic adaptation and antioxidant defense in IPF lungs.

To provide complementary cell-type-resolved information, we subsequently analyzed an independent single-cell RNA-seq dataset (GSE136831) of lung tissues from controls and IPF patients; from this analysis emerged that only 35.9% of fibrotic fibroblasts express ASAH1, consequently, the absence/inactivity of ceramidase, condition that characterize 64.1% of fibroblasts from IPF patients we analyzed, could promote a pathological/proliferative/pro-fibrotic phenotype of fibroblasts associated to an anti-oxidant system impairment. Consistent with the metabolomic findings, we found that ASAH1 positive fibroblasts in lung tissues of IPF patients showed enrichment of pathways related to oxidoreductase activity compared with ASAH1 negative fibroblasts, consistent with differences in transcriptional programs involved in redox adaptation and homeostasis. In addition, differential gene expression analysis revealed increased expression of several reported negative regulators of fibroblast-to-myofibroblast transition (FMT), including CITED2, SFRP4, SFRP1, RARRES1, SDC2 and THY. These transcriptional associations are consistent with the possibility that ASAH1 positive fibroblasts display molecular features associated with a less pro-fibrotic phenotype. Importantly, the single-cell transcriptomic analysis is observational and therefore cannot establish whether ASAH1 expression directly regulates these transcriptional programs. Rather, it provides human tissue-based evidence that complements the pharmacological findings obtained in cultured fibroblasts.

Interestingly, ceramidase is a key enzyme that catabolizes ceramide into sphingosine, which can subsequently be phosphorylated to generate S1P, a lipid mediator implicated in myofibroblasts activation and fibrosis progression (Donati et al., 2021). In our experimental model, hypoxia induced an increase in the expression of the S1P transporter Spinster homolog 2 (SPNS2) in fibroblasts (**Supplementary Figure 2G**), which was reduced following ceramidase inhibition. These findings are consistent with the possibility that ceramidase activity may be associated with the maintenance of a less activated fibroblast phenotype under hypoxic conditions, while potentially contributing to S1P synthesis and export, facilitating its known pro-fibrotic effect acting on the surrounding cells. The mechanism through which ceramidase promotes the metabolic shift necessary for the adaptation, remains to be investigated.

Several limitations should be acknowledged. The metabolic alterations identified in this study were obtained using pharmacological inhibition of ceramidase, and complementary genetic approaches will be valuable to further define the specific contribution of ASAH1. Moreover, the transcriptomic analyses are observational and identify associations rather than causal relationships. Although the pseudobulk strategy substantially reduced the impact of patients’ heterogeneity and transcript dropout, additional mechanistic studies will be required to establish how ASAH1 regulates fibroblast metabolism and activation *in vivo*. It is important to note that the pathway enrichment analysis was performed using metabolites selected on the basis of fold change rather than metabolite-level statistical significance. Therefore, individual metabolites contributing to the enrichment may reflect biological or analytical variability. Accordingly, these pathway-level results were considered exploratory and hypothesis-generating and were not used as standalone evidence for mechanistic conclusions. Rather, they were interpreted together with specific metabolite-level observations and independent transcriptomic and functional data. Moreover, validation in experimental models of pulmonary fibrosis and in human fibrotic tissues will be essential to determine the physiological relevance of these findings. Despite these limitations, the convergence of metabolic, redox, phenotypic and human transcriptomic observations provides a coherent framework linking acid ceramidase-dependent sphingolipid metabolism with fibroblast adaptation to hypoxic stress. Importantly, the pharmacological data identify metabolic and phenotypic changes associated with acid ceramidase perturbation, while independent bulk and single-cell analyses of human IPF lungs demonstrate that ASAH1 expression is associated with hypoxia-, oxidative-stress- and fibroblast activation-related transcriptional programs. Thus, our findings identify ASAH1 as a plausible candidate modulator of fibroblast metabolic adaptation and phenotypic remodeling in the hypoxic fibrotic lung.

In conclusion, our study provides pharmacological evidence that perturbation of acid ceramidase is associated with metabolic, redox and phenotypic changes in hypoxic fibroblasts, complemented by associative evidence from bulk and single-cell transcriptomic analyses of IPF lungs. While whole-lung transcriptomic correlations should be interpreted cautiously because of differences in cellular composition and potential clinical heterogeneity, the single-cell analysis provides additional evidence linking ASAH1 expression to specific transcriptional programs within IPF fibroblasts. Together, these findings identify ASAH1 as a candidate regulator of fibroblast adaptation to hypoxic stress and provide a rationale for future mechanistic studies using genetic and biochemical approaches.

## Data Availability

The datasets presented in this study can be found in online repositories. The names of the repository/repositories and accession number(s) can be found in the article/**Supplementary Material**.
