# Supplementary material for: Ceramidase is involved in metabolic reprogramming of fibroblasts under hypoxia conditions
Source: Front Physiol. 2026 Sep 30;17:1914022. doi: 10.3389/fphys.2026.1914022 (PMC13661358; doi:10.3389/fphys.2026.1914022)
Supplement: Supplementary file 1 [file SupplementaryFile1.docx]

Supplementary Material

**Supplementary Figure 1.** **A)** Mainly enriched pathways in metabolic reprogramming of hypoxia- compared to normoxia-exposed human fibroblasts; metabolites involved in antioxidant mechanisms, are marked with the violet star. **B)** Hypoxic (blue line) and normoxic (red line) fibroblasts viability. Data are presented as absorbance (550 nm) versus time treatment, and expressed as mean and error ± SD. **C)** Reduced (GSH) vs oxidative glutathione (glutathione disulfide, GSSG) relation in hypoxic fibroblasts. Human fibroblasts were exposed to hypoxia for 24 and 72 hours and an untargeted metabolomic analysis was performed; resulting data were analyzed with MetaboAnalyst 6.0 tool and the abundance of metabolites between two experimental conditions (hypoxia vs. normoxia) was evaluated. Data are expressed as Log2 Fold Change (FC) of GSH (black line) and GSSG (red line) in hypoxic compared to normoxic cells. **D)** Viability of fibroblasts exposed to hypoxia in absence (CTR_Hypoxia; light blue line) and in presence of ceramidase inhibitor (D-Nmappd_Hypoxia; dark blue line). Data are presented as absorbance (550 nm) versus time treatment, and expressed as mean and error ± SD. **E)** Enriched (top 25) metabolic pathways in D-Nmappd_hypoxia- compared to CTR_hypoxia-exposed fibroblasts. Human fibroblasts were exposed to hypoxia for 24 hours in presence of a specific inhibitor of ceramidase (D-Nmappd 5µM) and an untargeted metabolomic analysis was performed; resulting data were analyzed with MetaboAnalyst 6.0 tool and a metabolite set enrichment analysis (MSEA) was carried out. Dot plot of the enrichment analysis results of D-Nmappd_hypoxic compared to CTR_hypoxic fibroblasts. The size of the circles per metabolite set represents the Enrichment Ratio and the color represents the p value. Reduced (GSH) (**F**) vs oxidative glutathione (glutathione disulfide, GSSG) (**G**) in CTR_hypoxic fibroblasts compared to D-Nmappd_hypoxic fibroblasts. After an untargeted metabolomic analysis, the abundance of metabolites between two experimental conditions (CTR_hypoxic vs D-Nmappd_hypoxic and viceversa) was evaluated. Data are expressed as Log2 Fold Change (FC) of GSH (black vs green ine) and GSSG (black vs blue line) in CTR_hypoxic vs D-Nmappd_hypoxic, respectively, and viceversa.

**Supplementary Figure 2.** Human fibroblasts were exposed to hypoxia, in presence of ceramidase inhibitor (D-Nmappd, 5 µM); the percentage of cells positive to TMRE (**A**), Mitosox (**B**), Fibroblast Activation Protein (FAP) and Alpha Smooth Muscle Actin (αSMA) (**F**) and Spinster homolog 2 (SPNS2) (**G**) was evaluated by flow cytometry analysis, in normoxic (light red box) and hypoxic (light blue box) condition. Data are reported as median and represented as box plots with individual data points*.* Statistical differences were assessed through ordinary ONE-Way ANOVA, followed by Sidak post-test. Each dot represents an independent biological replicate. N-CTR: control normoxic fibroblasts; N-DNMAPPD: D-NMAPPD (ceramidase inhibitor) treated normoxic fibroblasts; H-CTR: control hypoxic fibroblasts; H-DNMAPPD: D-NMAPPD treated hypoxic fibroblasts. **C)** An untargeted metabolomic analysis was performed and Lactic acid was analyzed. Data are represented as box plots with individual data points. Statistical differences were assessed through ordinary ONE-Way ANOVA, followed by Sidak post-test. N-CTR: control normoxic fibroblasts; N-DNMAPPD: D-NMAPPD (ceramidase inhibitor) treated normoxic fibroblasts; H-CTR: control hypoxic fibroblasts; H-DNMAPPD: D-NMAPPD treated hypoxic fibroblasts. Transcriptomic analysis of single cell (sc) RNAseq dataset (GSE136831) from 28 controls and 32 idiopathic pulmonary fibrosis (IPF) patients (lung tissues) was performed. **D)** UMAP representing cell clusters, colored according to their identity using Leiden algorithm. **E)** Percentage expression of ceramidase transcripts (ASAH1) in fibroblasts cluster of controls and IPF patients from scRNAseq analysis (GSE136831).

**Supplementary Table 1.** Metabolite set enrichment analysis (MSEA) in hypoxia- compared to normoxia-24 hours exposed fibroblasts, performed by MetaboAnalyst 6.0 tool.

| **Enriched pathways** | **Total** | **Expected** | **Hits** | **Raw p** | **Holm p** | **FDR** |
| --- | --- | --- | --- | --- | --- | --- |
| Ammonia Recycling | 31 | 1.73 | 8 | 0.000166 | 0.0163 | 0.0163 |
| Purine Metabolism | 73 | 4.08 | 12 | 0.000378 | 0.0367 | 0.0185 |
| Urea Cycle | 28 | 1.56 | 6 | 0.00336 | 0.323 | 0.0908 |
| Glutathione Metabolism | 20 | 1.12 | 5 | 0.00371 | 0.352 | 0.0908 |
| Histidine Metabolism | 42 | 2.35 | 7 | 0.00678 | 0.638 | 0.133 |
| Warburg Effect | 57 | 3.19 | 8 | 0.011 | 1 | 0.18 |
| Glutamate Metabolism | 48 | 2.68 | 7 | 0.0142 | 1 | 0.197 |
| Methylhistidine Metabolism | 4 | 0.224 | 2 | 0.0171 | 1 | 0.197 |
| Lactose Synthesis | 19 | 1.06 | 4 | 0.0181 | 1 | 0.197 |
| Glucose-Alanine Cycle | 13 | 0.727 | 3 | 0.0317 | 1 | 0.311 |
| Beta-Alanine Metabolism | 34 | 1.9 | 5 | 0.0364 | 1 | 0.325 |
| Glycine and Serine Metabolism | 59 | 3.3 | 7 | 0.0406 | 1 | 0.331 |

Total: total number of metabolites belonging to that pathway in the reference database (Small Molecule Pathway Database, SMPDB); expected: the number of metabolites you would expect to find in that pathway by pure chance; hits: actual number of metabolites in your list that fall into that pathway; Raw p: uncorrected p-value of the statistical test; holm p: p-value corrected using the Holm–Bonferroni method; FDR: False Discovery Rate.

**Supplementary Table 2.** Metabolite set enrichment analysis (MSEA) in hypoxia- compared to normoxia-48 hours exposed fibroblasts, performed by MetaboAnalyst 6.0 tool.

| **Enriched pathways** | **Total** | **Expected** | **Hits** | **Raw p** | **Holm p** | **FDR** |
| --- | --- | --- | --- | --- | --- | --- |
| Ammonia Recycling | 31 | 1.14 | 6 | 0.000627 | 0.0615 | 0.0615 |
| Arginine and Proline Metabolism | 52 | 1.92 | 7 | 0.00208 | 0.201 | 0.0812 |
| Urea Cycle | 28 | 1.03 | 5 | 0.00276 | 0.265 | 0.0812 |
| Histidine Metabolism | 42 | 1.55 | 6 | 0.00331 | 0.315 | 0.0812 |
| Methylhistidine Metabolism | 4 | 0.148 | 2 | 0.0076 | 0.714 | 0.149 |
| Purine Metabolism | 73 | 2.7 | 7 | 0.0143 | 1 | 0.195 |
| Warburg Effect | 57 | 2.1 | 6 | 0.0152 | 1 | 0.195 |
| Methionine Metabolism | 42 | 1.55 | 5 | 0.0163 | 1 | 0.195 |
| Glycine and Serine Metabolism | 59 | 2.18 | 6 | 0.0179 | 1 | 0.195 |
| Spermidine and Spermine Biosynthesis | 18 | 0.665 | 3 | 0.0258 | 1 | 0.24 |
| Citric Acid Cycle | 32 | 1.18 | 4 | 0.027 | 1 | 0.24 |
| Mitochondrial Electron Transport Chain | 19 | 0.702 | 3 | 0.0299 | 1 | 0.24 |
| Nucleotide Sugars Metabolism | 20 | 0.739 | 3 | 0.0343 | 1 | 0.24 |
| Glutathione Metabolism | 20 | 0.739 | 3 | 0.0343 | 1 | 0.24 |
| Carnitine Synthesis | 22 | 0.812 | 3 | 0.044 | 1 | 0.287 |

Total: total number of metabolites belonging to that pathway in the reference database (Small Molecule Pathway Database, SMPDB); expected: the number of metabolites you would expect to find in that pathway by pure chance; hits: actual number of metabolites in your list that fall into that pathway; Raw p: uncorrected p-value of the statistical test; holm p: p-value corrected using the Holm–Bonferroni method; FDR: False Discovery Rate.

**Supplementary Table 3.** Metabolite set enrichment analysis (MSEA) in hypoxia- compared to normoxia-72 hours exposed fibroblasts, performed by MetaboAnalyst 6.0 tool.

| **Enriched pathways** | **Total** | **Expected** | **Hits** | **Raw p** | **Holm p** | **FDR** |
| --- | --- | --- | --- | --- | --- | --- |
| Ammonia Recycling | 31 | 0.99 | 10 | 6.92E-09 | 6.78E-07 | 6.78E-07 |
| Glycine and Serine Metabolism | 59 | 1.88 | 10 | 5.46E-06 | 0.000529 | 0.000247 |
| Glutamate Metabolism | 48 | 1.53 | 9 | 7.56E-06 | 0.000726 | 0.000247 |
| Urea Cycle | 28 | 0.894 | 7 | 1.27E-05 | 0.0012 | 0.000298 |
| Arginine and Proline Metabolism | 52 | 1.66 | 9 | 1.52E-05 | 0.00143 | 0.000298 |
| Histidine Metabolism | 42 | 1.34 | 7 | 0.00021 | 0.0196 | 0.00343 |
| Glutathione Metabolism | 20 | 0.639 | 5 | 0.000266 | 0.0245 | 0.00372 |
| Beta-Alanine Metabolism | 34 | 1.09 | 6 | 0.000466 | 0.0424 | 0.0057 |
| Warburg Effect | 57 | 1.82 | 7 | 0.00147 | 0.132 | 0.016 |
| Nucleotide Sugars Metabolism | 20 | 0.639 | 4 | 0.0029 | 0.258 | 0.0272 |
| Malate-Aspartate Shuttle | 10 | 0.319 | 3 | 0.00305 | 0.269 | 0.0272 |
| Nicotinate and Nicotinamide Metabolism | 35 | 1.12 | 5 | 0.00395 | 0.344 | 0.0316 |
| Carnitine Synthesis | 22 | 0.703 | 4 | 0.00419 | 0.36 | 0.0316 |
| Methylhistidine Metabolism | 4 | 0.128 | 2 | 0.0057 | 0.484 | 0.0399 |
| Glucose-Alanine Cycle | 13 | 0.415 | 3 | 0.00681 | 0.572 | 0.0445 |
| Cysteine Metabolism | 26 | 0.83 | 4 | 0.00782 | 0.649 | 0.0479 |
| Pyruvate Metabolism | 47 | 1.5 | 5 | 0.0142 | 1 | 0.0808 |
| Alanine Metabolism | 17 | 0.543 | 3 | 0.0148 | 1 | 0.0808 |
| Citric Acid Cycle | 32 | 1.02 | 4 | 0.0164 | 1 | 0.0847 |
| Amino Sugar Metabolism | 33 | 1.05 | 4 | 0.0183 | 1 | 0.0853 |
| Gluconeogenesis | 33 | 1.05 | 4 | 0.0183 | 1 | 0.0853 |
| Mitochondrial Electron Transport Chain | 19 | 0.607 | 3 | 0.0203 | 1 | 0.0863 |
| Lactose Synthesis | 19 | 0.607 | 3 | 0.0203 | 1 | 0.0863 |
| Aspartate Metabolism | 35 | 1.12 | 4 | 0.0224 | 1 | 0.0913 |
| Threonine and 2-Oxobutanoate Degradation | 20 | 0.639 | 3 | 0.0233 | 1 | 0.0914 |
| Transfer of Acetyl Groups into Mitochondria | 22 | 0.703 | 3 | 0.0302 | 1 | 0.112 |
| Homocysteine Degradation | 9 | 0.287 | 2 | 0.0309 | 1 | 0.112 |
| Glycolysis | 23 | 0.735 | 3 | 0.0339 | 1 | 0.119 |
| Pyruvaldehyde Degradation | 10 | 0.319 | 2 | 0.0379 | 1 | 0.124 |
| Estrone Metabolism | 24 | 0.766 | 3 | 0.038 | 1 | 0.124 |
| Methionine Metabolism | 42 | 1.34 | 4 | 0.0408 | 1 | 0.129 |
| Oxidation of Branched Chain Fatty Acids | 26 | 0.83 | 3 | 0.0467 | 1 | 0.139 |
| Phytanic Acid Peroxisomal Oxidation | 26 | 0.83 | 3 | 0.0467 | 1 | 0.139 |

Total: total number of metabolites belonging to that pathway in the reference database (Small Molecule Pathway Database, SMPDB); expected: the number of metabolites you would expect to find in that pathway by pure chance; hits: actual number of metabolites in your list that fall into that pathway; Raw p: uncorrected p-value of the statistical test; holm p: p-value corrected using the Holm–Bonferroni method; FDR: False Discovery Rate.

**Supplementary Table 4.** Metabolite set enrichment analysis (MSEA) in D-Nmappd_hypoxia- compared to CTR_hypoxia-48 hours exposed fibroblasts, performed by MetaboAnalyst 6.0 tool.

| **Enriched pathways** | **Total** | **Expected** | **Hits** | **Raw p** | **Holm p** | **FDR** |
| --- | --- | --- | --- | --- | --- | --- |
| Purine Metabolism | 73 | 3.64 | 11 | 0.000537 | 0.0526 | 0.0526 |
| Methionine Metabolism | 42 | 2.1 | 7 | 0.00353 | 0.343 | 0.173 |
| Arginine and Proline Metabolism | 52 | 2.59 | 7 | 0.0119 | 1 | 0.245 |
| Methylhistidine Metabolism | 4 | 0.2 | 2 | 0.0137 | 1 | 0.245 |
| Glutathione Metabolism | 20 | 0.998 | 4 | 0.0147 | 1 | 0.245 |
| Histidine Metabolism | 42 | 2.1 | 6 | 0.015 | 1 | 0.245 |
| Carnitine Synthesis | 22 | 1.1 | 4 | 0.0206 | 1 | 0.287 |
| Beta-Alanine Metabolism | 34 | 1.7 | 5 | 0.0234 | 1 | 0.287 |
| Oxidation of Branched Chain Fatty Acids | 26 | 1.3 | 4 | 0.0364 | 1 | 0.396 |
| Urea Cycle | 28 | 1.4 | 4 | 0.0463 | 1 | 0.423 |
| Beta Oxidation of Very Long Chain Fatty Acids | 17 | 0.848 | 3 | 0.0485 | 1 | 0.423 |

Total: total number of metabolites belonging to that pathway in the reference database (Small Molecule Pathway Database, SMPDB); expected: the number of metabolites you would expect to find in that pathway by pure chance; hits: actual number of metabolites in your list that fall into that pathway; Raw p: uncorrected p-value of the statistical test; holm p: p-value corrected using the Holm–Bonferroni method; FDR: False Discovery Rate.

**Supplementary Table 5.** Metabolite set enrichment analysis (MSEA) in D-Nmappd_hypoxia- compared to CTR_hypoxia-72 hours exposed fibroblasts, performed by MetaboAnalyst 6.0 tool.

| **Enriched pathways** | **Total** | **Expected** | **Hits** | **Raw p** | **Holm p** | **FDR** |
| --- | --- | --- | --- | --- | --- | --- |
| Ammonia Recycling | 31 | 1.39 | 10 | 2.64E-07 | 2.59E-05 | 2.59E-05 |
| Urea Cycle | 28 | 1.26 | 9 | 1.20E-06 | 0.000116 | 5.88E-05 |
| Aspartate Metabolism | 35 | 1.57 | 8 | 8.30E-05 | 0.00797 | 0.00271 |
| Arginine and Proline Metabolism | 52 | 2.34 | 9 | 0.000285 | 0.027 | 0.00643 |
| Histidine Metabolism | 42 | 1.89 | 8 | 0.000328 | 0.0308 | 0.00643 |
| Warburg Effect | 57 | 2.56 | 8 | 0.00275 | 0.256 | 0.0449 |
| Purine Metabolism | 73 | 3.28 | 9 | 0.00367 | 0.338 | 0.0512 |
| Glutamate Metabolism | 48 | 2.16 | 7 | 0.00418 | 0.38 | 0.0512 |
| Alanine Metabolism | 17 | 0.763 | 4 | 0.00551 | 0.496 | 0.0577 |
| Thiamine Metabolism | 9 | 0.404 | 3 | 0.00589 | 0.524 | 0.0577 |
| Glycine and Serine Metabolism | 59 | 2.65 | 7 | 0.0133 | 1 | 0.118 |
| Beta-Alanine Metabolism | 34 | 1.53 | 5 | 0.0153 | 1 | 0.125 |
| Glucose-Alanine Cycle | 13 | 0.584 | 3 | 0.0177 | 1 | 0.133 |
| Cysteine Metabolism | 26 | 1.17 | 4 | 0.0257 | 1 | 0.18 |
| Phenylalanine and Tyrosine Metabolism | 27 | 1.21 | 4 | 0.0292 | 1 | 0.191 |
| Mitochondrial Electron Transport Chain | 19 | 0.853 | 3 | 0.0496 | 1 | 0.247 |

Total: total number of metabolites belonging to that pathway in the reference database (Small Molecule Pathway Database, SMPDB); expected: the number of metabolites you would expect to find in that pathway by pure chance; hits: actual number of metabolites in your list that fall into that pathway; Raw p: uncorrected p-value of the statistical test; holm p: p-value corrected using the Holm–Bonferroni method; FDR: False Discovery Rate.
